# Adaptation and Validation of the Spanish Version of the Smartphone Application-Based Addiction Scale (SABAS)

**DOI:** 10.3390/bs15040496

**Published:** 2025-04-08

**Authors:** Sergio Hidalgo-Fuentes, Isabel Martínez-Álvarez, Fátima Llamas-Salguero, Miriam Villaseñor-León

**Affiliations:** 1 Facultad de Psicología y Logopedia, Universitat de València, Av. Blasco Ibáñez 21, 46010 Valencia, Spain; 2 Facultad de Ciencias de la Educación, Universidad a Distancia de Madrid (UDIMA), Vía de Servicio A-6 15, 28400 Collado Villalba, Spain; isabel.martinez.al@udima.es; 3 Facultad de Educación y Psicología, Universidad de Extremadura, Av. de Elvas, S/n, 06006 Badajoz, Spain; fatimalls@unex.es; 4 Facultad de Educación y Psicología, Universidad Francisco de Vitoria, M-515, km 1.800, 28223 Pozuelo de Alarcón, Spain; 9000266@alumnos.ufv.es

**Keywords:** smartphone addiction, problematic smartphone use, validity, reliability, psychometric properties

## Abstract

Smartphone addiction is very prevalent among university students and can negatively impact both their physical and mental health. This study aimed to translate the smartphone application-based addiction scale (SABAS) into Spanish and evaluate its psychometric properties among Spanish university students. A total of 297 university students (222 women and 75 men) participated in this study (*M* age = 20.88, *SD* = 4.58) by completing a sociodemographic questionnaire, the smartphone addiction scale-short version, the depression, anxiety, and stress scale-21, the fear of missing out scale, and the SABAS. The SABAS was translated into Spanish using the forward-backward method. The factor structure was analyzed using confirmatory factor analysis, while reliability was assessed with Cronbach’s alpha and McDonald’s omega coefficients. The SABAS showed acceptable reliability (α = 0.72; ω = 0.73), and all of its items were kept. Factor analysis revealed that the Spanish version of the SABAS was unifactorial and demonstrated excellent validity (χ^2^ = 10.285; *p* = 0.246; RMSEA = 0.034; CFI = 0.991; SRMR = 0.030). Significant associations were also observed between the SABAS score and the number of hours of smartphone use, fear of missing out, and psychological distress. Despite its brevity, the Spanish version of the SABAS provides good validity and reliability. Therefore, it can be used as a tool for screening smartphone addiction in Spanish university students.

## 1. Introduction

Over the past decades, the popularity of smartphones has grown steadily, reaching 4.247 billion users worldwide in 2024, with projections estimating over 6 billion users by 2029 (Statista, 2024). Unlike traditional mobile phones, which were limited to calls and SMS messages, smartphones offer a wide range of functions, such as access to email, social media, music, online shopping, video games, and internet browsing. These features have made smartphones indispensable devices in daily life, transforming how people interact, work, access information, and enjoy entertainment. It is estimated that people use their smartphones for an average of 171 min daily, checking their devices 47 times a day (Turner, 2024). In Spain, 99.5% of households have one or more smartphones, and up to 69.6% of children under the age of 15 use a smartphone (INE, 2024).

Despite the benefits of smartphone use in areas such as education and health (Banskota et al., 2020; Chandran et al., 2022; Moses et al., 2021; Timmers et al., 2020), excessive and uncontrolled use can lead to smartphone addiction. Smartphone addiction is a compulsive behavior characterized by physiological, psychological, emotional, and social dysfunctions resulting from excessive dependence on smartphones. It is primarily manifested through a lack of self-control and excessive device use (Chóliz, 2010; Y. H. Lin et al., 2014). Although widely used in scientific literature, the concept of smartphone addiction is rejected by some researchers, who argue against the excessive pathologization of contemporary lifestyle elements, the lack of clinical and longitudinal studies, and the non-recognition of this behavior as an addictive disorder in major diagnostic manuals (Billieux et al., 2015a; Billieux et al., 2015b; Kardefelt-Winther, 2017; Lowe-Calverley & Pontes, 2020; Montag et al., 2021; Panova & Carbonell, 2018). These researchers advocate for alternative terms, such as problematic smartphone use. Despite the controversy surrounding its terminology, the terms smartphone addiction and problematic smartphone use are commonly used interchangeably (Busch & McCarthy, 2021; Thomée, 2018), and the term addiction is frequently employed in the names of many scales that assess problematic smartphone use. In the present study, we have adopted the term “smartphone addiction” due to the theoretical foundation of the SABAS, which was developed using Griffiths’ (2005) components model of addiction.

Smartphone addiction has been associated with various negative consequences in personal, social, and academic domains. These adverse effects include anxiety (Ratan et al., 2021), depression (Zhong et al., 2022), stress (Osorio-Molina et al., 2021), poor sleep quality (Leow et al., 2023), loneliness (Ge et al., 2023), fear of missing out (Elhai et al., 2020), phubbing (Arenz & Schnauber-Stockmann, 2024), academic procrastination (Hidalgo-Fuentes, 2022), low academic performance (Sunday et al., 2021), and, overall, a lower perception of well-being and quality of life (Ding et al., 2022). In addition to these negative consequences, smartphone addiction exhibits a high prevalence rate, affecting 26.99% of the general population (Meng et al., 2022), with a significant increase in recent years (Olson et al., 2022). Moreover, the prevalence of smartphone addiction is particularly high among university students, reaching 67% (Candussi et al., 2023), underscoring the need for further research in this context. While some recent studies have not found differences in the prevalence of smartphone addiction based on sex (Shi et al., 2023; Yue et al., 2023), numerous studies have reported a higher prevalence among women (Horwood & Anglim, 2018; Lee et al., 2018; Luk et al., 2018; Mahajan et al., 2017; Servidio et al., 2023).

The increasing interest in studying smartphone addiction in recent years has led to the development of at least 78 scales designed to assess this phenomenon since 2004 (Harris et al., 2020b), such as the smartphone addiction scale (Kwon et al., 2013b), the Smartphone Addiction Inventory (Y. H. Lin et al., 2014), the Mobile Phone Addiction Index (L. Leung, 2008), and the smartphone application-based addiction scale (SABAS) (Csibi et al., 2016), which is the focus of this study. The SABAS was developed based on the addiction components model of Griffiths (2005), which includes the following components: salience, tolerance, mood modification, relapse, withdrawal, and conflict, providing a solid theoretical foundation for its use in assessing smartphone addiction. The psychometric properties of the original SABAS version have proven to be robust, demonstrating a single-factor structure, adequate internal consistency (α = 0.82), and convergent validity with the Smartphone Addiction Inventory (Y. H. Lin et al., 2014), supporting its reliability and validity as a tool for assessing the risk of smartphone addiction. In addition to its strong psychometric properties, the brevity of the scale, comprising only six items, facilitates its application and significantly reduces the time required for completion. Subsequent adaptations to various languages and cultures have confirmed the scale’s single-factor structure and reliability, reinforcing its utility across different contexts and populations (Chen et al., 2020; Csibi et al., 2018; Islam et al., 2021; H. Leung et al., 2020; C. Y. Lin et al., 2019; Mason et al., 2022; Nurmala et al., 2022; Ruckwongpatr et al., 2024; Soraci et al., 2021; Tung et al., 2022; Vally & Alowais, 2020; Vujić et al., 2023; Yam et al., 2018). Additionally, a meta-analysis on the generalization of reliability evaluated 31 studies using the SABAS, finding a combined reliability of α = 0.81 (Hidalgo-Fuentes, 2023b). Although the SABAS has been translated and validated in numerous languages, to the best of our knowledge, no psychometric validation of this scale has been conducted in the Spanish context.

Therefore, the objective of this study was to analyze the psychometric properties of the Spanish version of the SABAS in Spanish university students. First, construct validity was evaluated through confirmatory factor analysis (CFA). Second, the reliability of the scale was assessed using internal consistency indices. Third, the convergent validity of the total SABAS scores was examined using other related measures. Finally, floor and ceiling effects were analyzed.

## 2. Materials and Methods

### 2.1. Participants and Procedure

An online survey was designed using Google Forms to collect the data necessary for the study. The inclusion criteria for participants were being a Spanish university student and owning a smartphone. The survey was distributed to students in Early Childhood Education and Primary Education programs at a Spanish public university during their class time in November 2024, after obtaining authorization from their professors. Participation was entirely voluntary, and students did not receive any incentives for taking part in the research.

The survey included two attention check questions, randomly embedded, designed to identify inattentive responses that could distort the study’s results and threaten its validity (Abbey & Meloy, 2017). Only participants who correctly answered both attention check questions were included in the analysis. The questionnaire was distributed to 404 students, of whom 362 initially participated (response rate: 89.7%). However, 17.96% (*n* = 65) were excluded for failing to answer the attention check questions correctly. This rate of inattentive responses falls within the typical range reported in online survey research (Nichols & Edlund, 2020; Ward et al., 2017). The removal of participants who do not correctly answer attention check questions is the most common strategy to ensure data quality in surveys, as it significantly improves the validity of the results (Ward & Meade, 2023). To assess the quality of the remaining data, the $\hat{Q}$ data quality index was used, a measure calculated by excluding participants who provided random or inattentive responses, providing an accurate estimate of the proportion of participants who, after data cleaning, completed the survey attentively and conscientiously (Taplin, 2024). The formula for calculating $\hat{Q}$ is (*x*/*n* − *p*)/(*x*/*n* × (1 − *p*)), where *x* is the number of participants who answered the attention check question correctly, *n* is the total number of participants, and *p* is the probability of a correct response in the case of random guessing, determined by the number of options in each attention check question. In this study, one of the attention check questions had four possible options (with a probability of correct guessing *p* = 0.25), while the other had seven options (with a probability of correct guessing *p* = 0.14), so after calculating $\hat{Q}$ for the participants who passed both attention check questions, the following results were obtained: For the first attention check question (with four options), out of a total of 362 participants, 325 answered correctly, resulting in a proportion of 0.90 (*x*/*n* = 325/362); the probability of correct random guessing *p* was 0.25, which yielded a $\hat{Q}$ index of 0.95 for the participants who answered this question correctly. For the second attention check question (with seven options), 319 of the 362 participants answered correctly, resulting in a proportion of 0.88 (*x*/*n* = 319/362); the probability of correct random guessing *p* was 0.14, leading to a $\hat{Q}$ index of 0.98. Therefore, an overall $\hat{Q}$ index was obtained, reflecting a high quality of the remaining data, with an average value of approximately 0.97, indicating that 97% of the remaining participants completed the survey attentively.

The final sample comprised 297 students (222 women and 75 men) from a Spanish public university. The participants’ ages ranged from 17 to 54 years, with a mean of 20.88 years (*SD* = 4.58). Since a sample size of 5 to 10 observations per item included in a factor analysis is recommended (Comrey & Lee, 1992), and the scale under validation consists of six items, the sample size far exceeded this criterion.

Regarding ethical considerations, the study was approved by the Bioethics and Biosafety Commission of the University of Extremadura, under reference number 296/2024. The first page of the questionnaire provided detailed information about the study’s objectives and its anonymous and voluntary nature, and participants gave informed consent before starting the survey through a specific item included in the questionnaire.

### 2.2. Translation and Adaptation

After obtaining permission from the authors of the scale, the English version of the SABAS was translated and adapted following a standardized approach for test translation and adaptation (Muñiz et al., 2013). The SABAS was translated using a forward-translation and back-translation process. The Spanish translation was conducted by two translators, while the back-translation into English was carried out by a single translator, adhering to the guidelines established by Hall et al. (2017). The translation from English to Spanish was performed by two Spanish-speaking psychologists with proficiency in English and familiarity with the scale’s subject matter and terminology. Discrepancies between the two translated versions were resolved through discussion among the translators, resulting in a harmonized version. This harmonized version was then back-translated into English by an independent professional translator, a native English speaker with no prior knowledge of the scale or its subject matter. The original English version and the back-translation were compared to ensure that no significant aspects had been altered during the translation process and to confirm conceptual equivalence. Finally, a pilot test was conducted with a group of 12 university students (66.67% women, *M* age = 21.5 years, *SD* = 2.06) to identify potential ambiguities in the wording and ensure that the target audience could easily understand the scale. This sample size is considered sufficient for an initial evaluation of the translation’s comprehensibility (EORTC Quality of Life Group, 2017; Koller et al., 2007). The students rated the clarity of each item on a five-point scale ranging from 1 (very difficult to understand) to 5 (very easy to understand). Additionally, they provided feedback on the quality of the items. Most students found the items in the Spanish version of the SABAS to be very easy to understand; however, a few minor revisions were suggested and subsequently implemented.

### 2.3. Measurements

Sociodemographic data such as participants’ age and gender were collected. Participants were also asked about the number of daily hours spent using their smartphones.

Smartphone Application-Based Addiction Scale (SABAS; Csibi et al., 2016): This scale assesses the risk of smartphone addiction based on the components model of addiction proposed by Griffiths (2005). Items are rated on a 6-point Likert scale ranging from 1 (strongly disagree) to 6 (strongly agree). Scores range from 6 to 36 points, with higher scores indicating a greater risk of smartphone addiction.

Smartphone Addiction Scale-Short Version (SAS-SV; Kwon et al., 2013a): This is the short version of the SAS, developed to measure smartphone addiction (Kwon et al., 2013b). The scale consists of ten items with a 6-point Likert response format, ranging from 1 (strongly disagree) to 6 (strongly agree). Total scores range from 10 to 60, with higher scores associated with a higher prevalence of smartphone addiction. For this study, the Spanish adaptation by Lopez-Fernandez (2017), with a reliability of α = 0.81, was used.

Depression, Anxiety, and Stress Scale-21 (DASS-21; Lovibond & Lovibond, 1995): Depression and anxiety were assessed using the DASS-21, a tool designed to measure negative emotional states of depression, anxiety, and stress. Each state is evaluated through a subscale of seven items. Participants responded based on their emotional state during the past week using a 4-point Likert scale, ranging from 1 (did not apply to me at all) to 4 (applied to me very much, or most of the time). Higher scores indicate higher levels of depression, anxiety, and stress. In this study, the Spanish version adapted by Daza et al. (2002) was used. The depression subscale demonstrated a reliability of α = 0.86, the anxiety subscale α = 0.84, and the stress subscale α = 0.88.

Fear of Missing Out Scale (FoMO; Przybylski et al., 2013): This scale measures Fear of Missing Out (FoMO) through 10 items using a Likert-type response scale ranging from 1 (not at all true of me) to 5 (extremely true of me). Higher scores indicate higher levels of FoMO. The possible score range on the FoMO is 10 to 50. For this study, the Spanish adaptation by Gil et al. (2015), with a reliability of α = 0.85, was used.

### 2.4. Data Analysis

First, the univariate normality of the Spanish version of the SABAS was evaluated by analyzing the skewness and kurtosis values of each item. Items were considered normally distributed if their skewness values fell within the range of ±2 and kurtosis values within ±7 (Byrne, 2010; Hair et al., 2010). Multivariate normality was assessed using Mardia’s (1970) test.

The reliability of the Spanish version of the SABAS was examined through internal consistency, including Cronbach’s alpha, McDonald’s omega, corrected item–total correlations, alpha if item deleted, and omega if item deleted. Cronbach’s alpha and McDonald’s omega values of 0.70 or higher were considered indicative of acceptable reliability (DeVellis, 2016; Nunnally & Bernstein, 1994). Items were deemed appropriate if their corrected item–total correlation exceeded 0.30 and their removal did not increase Cronbach’s alpha or McDonald’s omega by 0.10 or more (Nunnally & Bernstein, 1994). Additionally, omega if item deleted was calculated to further assess the contribution of each item to the overall reliability of the scale.

Factorial validity was evaluated using a confirmatory factor analysis (CFA). A unifactorial structure has been consistently identified during the development of the original SABAS, as well as in its various adaptations. Since the objective of the present study was to validate this structure, it was deemed more appropriate to conduct a CFA to assess the fit of the factorial structure of the Spanish version of the SABAS, rather than using an Exploratory Factor Analysis (Knekta et al., 2019; Martínez et al., 2014). The robust maximum likelihood estimation method (MLR) was employed, suitable for data that do not meet multivariate normality assumptions, using the raw data as the basis. Model fit was evaluated with the following criteria (Hu & Bentler, 1999; Ullman, 2014): a non-significant chi-square, comparative fit index (CFI ≥ 0.95), root mean square error of approximation (RMSEA ≤ 0.06), and standardized root mean square residual (SRMR ≤ 0.08). Modification indices were examined to further investigate the structure of the Spanish version of the SABAS and potential sources of poor fit, improving the model fit by adding covariance paths between error terms where necessary. This means that we allowed the residuals of two items to correlate, which is appropriate when the items share similar content or are conceptually related. By allowing the error terms to correlate, we account for the possibility that the items may be influenced by common factors or unique influences not captured by the main latent variable.

Construct validity was assessed by evaluating the convergent validity of the Spanish SABAS with the total scores of the SAS-SV, the FoMO, and the depression, anxiety, and stress subscales of the DASS-21, as well as with the number of daily smartphone usage hours. Given that all test scores followed a normal distribution, Pearson’s bivariate correlations were used to examine these relationships. Correlations were interpreted based on the criteria by Gignac and Szodorai (2016), which classify correlations as small (0.10), moderate (0.20), or large (0.30 or higher).

Floor and ceiling effects were evaluated by calculating the percentage of participants who achieved the minimum and maximum possible scores, respectively. Floor or ceiling effects were considered present if more than 15% of participants obtained either extreme score on the test (McHorney & Tarlov, 1995).

Data were analyzed using RStudio (Version 2022.7.1.554), using the R (Version 4.2.1) programming language for statistical computing. We used packages lavaan v06.19 (Rosseel, 2012), psych v2.4.3 (Revelle, 2021) and MVN v5.9 (Korkmaz et al., 2014). The lavaan package was used for confirmatory factor analysis, MVN for assessing multivariate normality, and psych for calculating internal consistency, Pearson correlations for convergent validity, item descriptives, univariate normality, as well as for analyzing floor and ceiling effects.

## 3. Results

The normality of the items was evaluated using skewness and kurtosis indices, which remained within the range of ±2 for skewness and ±7 for kurtosis, indicating that the items were normally distributed (Table 1). However, when assessing multivariate normality using Mardia’s test, it was found that the data did not meet this assumption. Although the multivariate kurtosis statistic showed a result of Z = 1.64, *p* = 0.10, indicating no significant deviations in multivariate kurtosis, the multivariate skewness statistic resulted in χ^2^ = 154.98, *p* < 0.001, which signals a significant deviation in the symmetry of the data. Table 1 presents the characteristics of the sample, including demographic information and the scores of the tests employed, along with the distribution of daily hours spent using smartphones. Regarding the SABAS score based on gender, women scored 17.10 (*SD* = 5.33), while men scored 15.24 (*SD* = 4.19), with the difference being statistically significant (t = −3.09; *p* = 0.006). The sample has been classified according to the quartiles of the SABAS score: Q1 = 13, Q2 = 17, and Q3 = 20. Based on this classification, four groups were identified. The first group, consisting of participants with scores equal to or lower than 13 (Q1), represents 33.7% of the sample (100 participants). The second group, with scores between 14 and 17 (between Q1 and Q2), includes 27.6% (82 participants). The third group, with scores between 18 and 20 (between Q2 and Q3), comprises 20.9% (62 participants). Finally, the fourth group, made up of those with scores higher than 20 (Q3), accounts for 17.8% (53 participants). This classification provides insight into the distribution of the sample according to smartphone addiction levels and facilitates the analysis of differences among the varying degrees of addiction.

All items showed corrected item–total correlations exceeding the recommended value of 0.30 (Table 2). The reliability of the full scale, assessed using Cronbach’s alpha, was 0.72, and using McDonald’s omega, it was 0.73. Furthermore, the removal of any item did not lead to a significant change in Cronbach’s alpha or McDonald’s omega, which further supports the internal consistency and reliability of the instrument.

**Figure 1:**
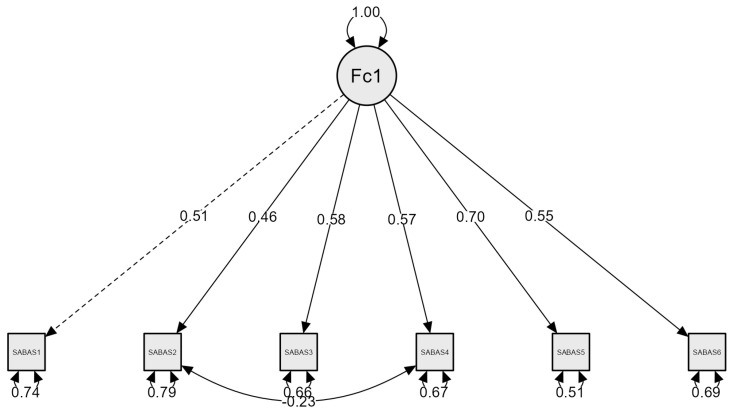
Factor structure of Model 2 (unidimensional with MI), with standardized parameters.

In the CFA, the fit of the unidimensional model observed in the original version of the SABAS and its various adaptations was compared to the data (Table 3). The CFA of the unidimensional version of the Spanish SABAS presented a significant chi-square (*p* = 0.020), but the other indices supported an acceptable fit of the model to the data (CFI = 0.957, RMSEA = 0.068, SRMR = 0.043). Although the fit could be considered adequate, the modification indices suggested that adding an error covariance between item 2 and item 4 would improve the model fit. Since these items had related content, a second model was tested, allowing for error covariance between the two items. Adding this modification improved the model, which then presented a non-significant chi-square and excellent fit indices (CFI = 0.991, RMSEA = 0.034, SRMR = 0.030). The corresponding path diagram is presented to visually illustrate the model and its structure (Figure 1). All items significantly loaded onto the latent variable. The factor loadings of the six items ranged from 0.46 to 0.70 (Table 4).

The construct validity of the Spanish version of the SABAS was supported by evidence of convergent validity with the SAS-SV (Table 5), one of the most commonly used instruments to assess the risk of smartphone addiction. The total score of the Spanish version of the SABAS also showed statistically significant correlations with the FoMO and the subscales of depression, anxiety, and stress from the DASS-21. Additionally, the SABAS correlated with the number of daily hours spent using smartphones by the participants. The correlations with the SAS-SV, FoMO, the anxiety subscale, and the number of daily hours spent using smartphones were high in intensity, while those with the depression and stress subscales were moderate in intensity.

In the Spanish version of the SABAS, no floor or ceiling effects were observed, as only 1.3% of participants obtained the minimum score and no participant obtained the maximum score.

## 4. Discussion

The aim of the present study was to translate and assess the psychometric properties of the Spanish version of the SABAS, a brief tool for assessing smartphone addiction, in Spanish university students. To the best of our knowledge, this is the first time the psychometric qualities of this scale have been examined in the Spanish context, representing a significant contribution to the literature on the assessment of smartphone addiction across different populations and cultures. The World Health Organization recognizes that the excessive use of electronic devices, including smartphones, can have negative consequences for public health, similar to disorders such as pathological gambling or substance use (World Health Organization, 2015). It emphasizes the importance of regularly collecting population-based data using standardized and validated instruments, with the aim of developing effective prevention strategies. This highlights the importance of validating scales with good psychometric properties and a solid theoretical foundation, ensuring the quality and accuracy of the data.

The results obtained from the CFA support the unidimensional model of the SABAS, a finding consistent with previous results from both the original version of the scale and its subsequent adaptations. This unidimensional model suggests that the scale consistently and coherently measures a single underlying construct: smartphone addiction. The fit indices obtained in the CFA were excellent, indicating that the model structure is highly suitable for the sample of Spanish university students, thus reinforcing the structural validity of the Spanish version of the SABAS. The results indicate that all standardized factor loadings are significant (*p* < 0.001) and above 0.40, suggesting that each item adequately contributes to measuring the latent construct. Specifically, the values range from 0.46 to 0.70, indicating a moderate to strong relationship between the items and the underlying factor. These findings support the validity of the proposed factor structure and suggest that the items consistently represent the theoretical concept being measured. In the final model, the error covariance between items 2 and 4 was added, as they are related to the conflict and tolerance components of Griffiths’ (2005) model. The conflict and tolerance components may be bidirectionally related, as increased usage time associated with tolerance may lead to interpersonal conflicts, while these conflicts can intensify smartphone use as an emotionally maladaptive coping strategy (Huang et al., 2023; Wegmann et al., 2023). However, conflicts arising from excessive use could, in some cases, reduce tolerance by raising awareness of its negative consequences. Therefore, this modification was not only included due to the improvement in the model parameters but also because it has consistent theoretical support, as the interaction between conflict and tolerance reflects dynamics characteristic of addictive behaviors.

The internal consistency of the Spanish version of the SABAS showed a Cronbach’s alpha of 0.72 and a McDonald’s omega of 0.73, indicating adequate reliability (DeVellis, 2016; Nunnally & Bernstein, 1994). Although these values are considered acceptable, they are lower than those reported in other adaptations of the scale. Possible reasons for this could include cultural differences in response patterns, variations in sample characteristics, or the specific context in which the scale was applied in this study. Corrected item–total correlations were calculated to determine the relationship between the scores obtained from the SABAS items and the total score of the scale, ranging from 0.34 to 0.60. This demonstrates that all items have a significant relationship with the total score of the scale, as they exceed the recommended criterion of 0.30 (Nunnally & Bernstein, 1994).

The convergent validity of the SABAS was confirmed through its high correlation with the SAS-SV, one of the most widely used instruments for assessing smartphone addiction. This finding is consistent with previous studies that have reported a strong correlation between both measures (Al-Qarni & El Keshky, 2022; Harris et al., 2020a; Vujić et al., 2023), reaffirming the capacity of the SABAS to adequately capture this construct in the Spanish university population. The total score of the SABAS has also shown positive correlations of moderate to high magnitude with the three subscales of the DASS-21, which aligns with the results of several meta-analyses and reviews that have found smartphone addiction to be associated with anxiety, stress, and depression (Elhai et al., 2019; Ran et al., 2022; Ratan et al., 2021; Yang et al., 2020). However, the direction of this relationship remains a subject of debate, as some studies suggest that smartphone addiction increases the levels of these disorders (Coyne et al., 2019; Santander-Hernández et al., 2022), while other research indicates that psychological distress heightens the risk of developing smartphone addiction (Kim & Koh, 2018; Zhou et al., 2021). Additionally, a strong positive correlation has been found between the SABAS score and the FoMO score, which was expected given that a recent meta-analysis has shown a consistent relationship between fear of missing out and smartphone addiction (Hidalgo-Fuentes, 2023a). This suggests that individuals with high levels of fear of missing out are at greater risk of developing addictive behaviors related to smartphone use, particularly in connection with social use and the need to stay connected with others (Wolniewicz et al., 2018). These individuals tend to use their devices compulsively to alleviate the anxiety associated with disconnection, which in turn increases the risk of addictive behaviors, as captured by the SABAS. This finding aligns with the theoretical model proposed by Billieux et al. (2015a), which identifies the reassurance pathway as a route toward problematic smartphone use, characterized by a heightened need for connection and social security. Finally, the SABAS has also shown a positive correlation with the number of daily hours of smartphone use. While high smartphone usage time does not necessarily lead to negative effects (Olson et al., 2022), numerous studies have found a relationship between smartphone addiction and self-reported usage time (Andrade et al., 2020; Lopez-Fernandez, 2017; Nawaz et al., 2024; Nikolic et al., 2022; Randjelovic et al., 2021). Taken together, these findings are consistent with Griffiths’ (2005) components model of addiction, which emphasizes the interaction of psychological, emotional, and behavioral factors in the development of addiction.

Additionally, no floor or ceiling effects were observed, as the percentage of participants who obtained the lowest and highest scores did not exceed the 15% threshold (McHorney & Tarlov, 1995). This suggests that the test has adequate sensitivity to discriminate between different levels of the evaluated construct.

The results of the present study have several limitations that must be considered when interpreting the findings. First, participants were recruited using convenience sampling, and the sample showed a significant imbalance between women and men, which limits the generalization of the results. Despite the observed gender imbalance (74.75% women), this reflects a natural characteristic of the target population. According to data from the Integrated University Information System (SIIU) of the Ministry of Science, Innovation and Universities (2025), 77.2% of students in Early Childhood Education and Primary Education programs within the Spanish public university system are women. Even so, caution is recommended when extrapolating the findings. Future studies would benefit from employing probabilistic sampling methods to increase the representativeness of the sample, particularly by including participants from different universities. Second, all the tests used in this study were self-reported, which implies the possibility of response biases; however, the anonymous and voluntary nature of the survey helps mitigate this risk (Dodou & de Winter, 2014). Nevertheless, future studies could benefit from using passive monitoring techniques to objectively assess smartphone use. These techniques allow for the collection of accurate and ecologically valid data on smartphone users’ behavior, such as screen time, without relying on the active intervention of participants (Ryding & Kuss, 2020). Third, while the time participants spent on their smartphones daily was assessed, the specific uses of the technology were not considered. Therefore, future studies should investigate the specific online activities that university students engage in to assess their impact on the risk of smartphone addiction. Finally, this study did not evaluate the psychometric properties of the SABAS to the fullest extent. Future research would be relevant to examine the test–retest reliability of the SABAS in the Spanish population to ensure the temporal consistency of the instrument in this context, as well as to assess the measurement invariance of the scale across different groups (e.g., gender or age groups), which could not be performed due to the inadequate sample size for multigroup analysis (Meade, 2005).

Despite these limitations, this study represents the first Spanish translation and validation of the SABAS, providing a reliable and valid tool to assess the risk of smartphone addiction in Spanish university students. The validation of the Spanish version of SABAS addresses the need for a brief, theoretically well-grounded tool to assess the risk of smartphone addiction. SABAS is based on a solid theoretical framework that highlights the cognitive and behavioral components involved in problematic smartphone use, an approach that has proven relevant in previous studies. Moreover, its brevity, only six items, makes it an efficient option in contexts where response burden must be minimized without compromising its validity and reliability. Our findings support and complement previous research on the psychometric properties of this scale in samples from other cultures and countries. Furthermore, our results highlight important opportunities for future research, such as the validation of the SABAS in different samples (e.g., adolescents or clinical samples) and the evaluation of other psychometric properties, such as test–retest reliability, measurement invariance, and the use of the multitrait–multimethod approach to provide a more comprehensive assessment of the instrument.

## Figures and Tables

**Table 1:** Characteristics of the sample (*n* = 297).

Variables	*M*	*SD*	*n*	%
Age	20.88	4.58		
Gender				
Man			222	74.75
Woman			75	25.25
SABAS	16.63	5.12		
SAS-SV	25.43	8.21		
DASS-21 depression	3.73	4.30		
DASS-21 anxiety	4.09	4.39		
DASS-21 stress	5.90	4.98		
FoMO	21.81	7.06		
Daily hours spent using smartphones				
Less than 1 h			1	0.3
From 1 to 2 h			11	3.7
From 2 to 3 h			33	11.1
From 3 to 4 h			68	22.9
From 4 to 5 h			89	30
From 5 to 6 h			50	16.8
More than 6 h			45	15.2

**Table 2:** Item analysis and internal consistency of SABAS for Spanish university students.

Item	Statement	*M*	*SD*	Skew	Kurtosis	Corrected Item–Total Correlation	Cronbach’s Alpha If Item Deleted	McDonalds’s Omega If Item Deleted
1	ENG: My smartphone is the most important thing in my life ESP: Mi smartphone es la cosa más importante en mi vida	2.64	1.23	0.36	−0.41	0.42	0.69	0.70
2	ENG: Conflicts have arisen between me and my family (or friends) because of my smartphone use ESP: Mi uso del smartphone ha provocado discusiones con mi familia (o amigos)	2.35	1.36	0.89	−0.01	0.34	0.72	0.72
3	ENG: Preoccupying myself with my smartphone is a way of changing my mood (I get a buzz, or I can escape or get away, if I need to) ESP: Centrarme en mi smartphone es una forma de cambiar mi estado de ánimo (me anima, o puedo escapar o desconectarme si lo necesito)	3.41	1.39	−0.07	−0.87	0.50	0.67	0.69
4	ENG: Over time, I fiddle around more and more with my smartphone ESP: A lo largo del tiempo, jugueteo cada vez más con mi smartphone	3.04	1.34	0.16	−0.75	0.44	0.69	0.70
5	ENG: If I cannot use or access my smartphone when I feel like, I feel sad, moody, or irritable ESP: Me siento triste, de mal humor o irritable cuando no puedo usar mi smartphone	2.22	1.18	0.82	0.01	0.60	0.65	0.65
6	ENG: If I try to cut the time I use my smartphone, I manage to do so for a while, but then I end up using it as much or more than before ESP: Si intento reducir el tiempo que uso mi smartphone, lo consigo por un tiempo, pero después termino usándolo igual o más que antes	2.97	1.42	0.36	−0.63	0.47	0.68	0.69

Notes: *M* = mean; *SD* = standard deviation; ENG: original statement in English; ESP: adaptation in Spanish.

**Table 3:** Comparison of goodness-of-fit indices obtained through CFA.

Model	*χ* ^2^	*df*	*p*	RMSEA (90%IC)	SRMR	CFI
Model 1 (unidimensional)	19.725	9	0.020	0.068 [0.026; 0.110]	0.043	0.957
Model 2 (unidimensional with MI)	10.285	8	0.246	0.034 [0.000; 0.086]	0.030	0.991

Notes. *χ*^2^ = chi-square; *df* = degrees of freedom; RMSEA = root mean square error of approximation; SRMR = standardized root mean squared residual; CFI = comparative fit index; MI = modification index.

**Table 4:** Unstandardized and standardized loadings for the measurement model.

Item	Unstandardized Loading (SE)	*p*	Unstandardized Loading CI95%	Sandardized Loading (SE)	*p*	Standardized Loading CI95%
1	1.00		1.00; 1.00	0.51 (0.06)	<0.001	0.40; 0.62
2	1.00 (0.20)	<0.001	0.61; 1.39	0.46 (0.06)	<0.001	0.35; 0.58
3	1.29 (0.19)	<0.001	0.93; 1.66	0.59 (0.04)	<0.001	0.50; 0.67
4	1.22 (0.20)	<0.001	0.83; 1.61	0.57 (0.06)	<0.001	0.47; 0.68
5	1.32 (0.15)	<0.001	1.02; 1.62	0.70 (0.05)	<0.001	0.61; 0.79
6	1.25 (0.19)	<0.001	0.87; 1.63	0.55 (0.06)	<0.001	0.44; 0.67

Notes: SE = standard error.

**Table 5:** Correlation coefficients between the total score of the Spanish version of the SABAS and the scores of the SAS-SV, DASS-21, FoMO, and time of smartphone usage.

Variables	*r*	CI 95%	*p*
Daily hours spent using smartphones	0.327	[0.222; 0.425]	<0.001
SAS-SV	0.728	[0.669; 0.777]	<0.001
DASS-21 depression	0.279	[0.170; 0.380]	<0.001
DASS-21 anxiety	0.325	[0.219; 0.423]	<0.001
DASS-21 stress	0.277	[0.169; 0.379]	<0.001
FoMO	0.408	[0.308; 0.499]	<0.001

Notes: *r* = Pearson correlation; CI 95% = 95% confidence interval; *p* = significance level.

## Data Availability

The data presented in this study are available on request from the corresponding author.
